# Supplementary material for: pH Nonlinearly Dominates Soil Bacterial Community Assembly along an Altitudinal Gradient in Oak-Dominant Forests
Source: Microorganisms. 2024 Sep 11;12(9):1877. doi: 10.3390/microorganisms12091877 (PMC11434175; doi:10.3390/microorganisms12091877)
Supplement: Supplementary file 1 [file microorganisms-12-01877-s001.zip › microorganisms-3164381-supplementary.pdf]

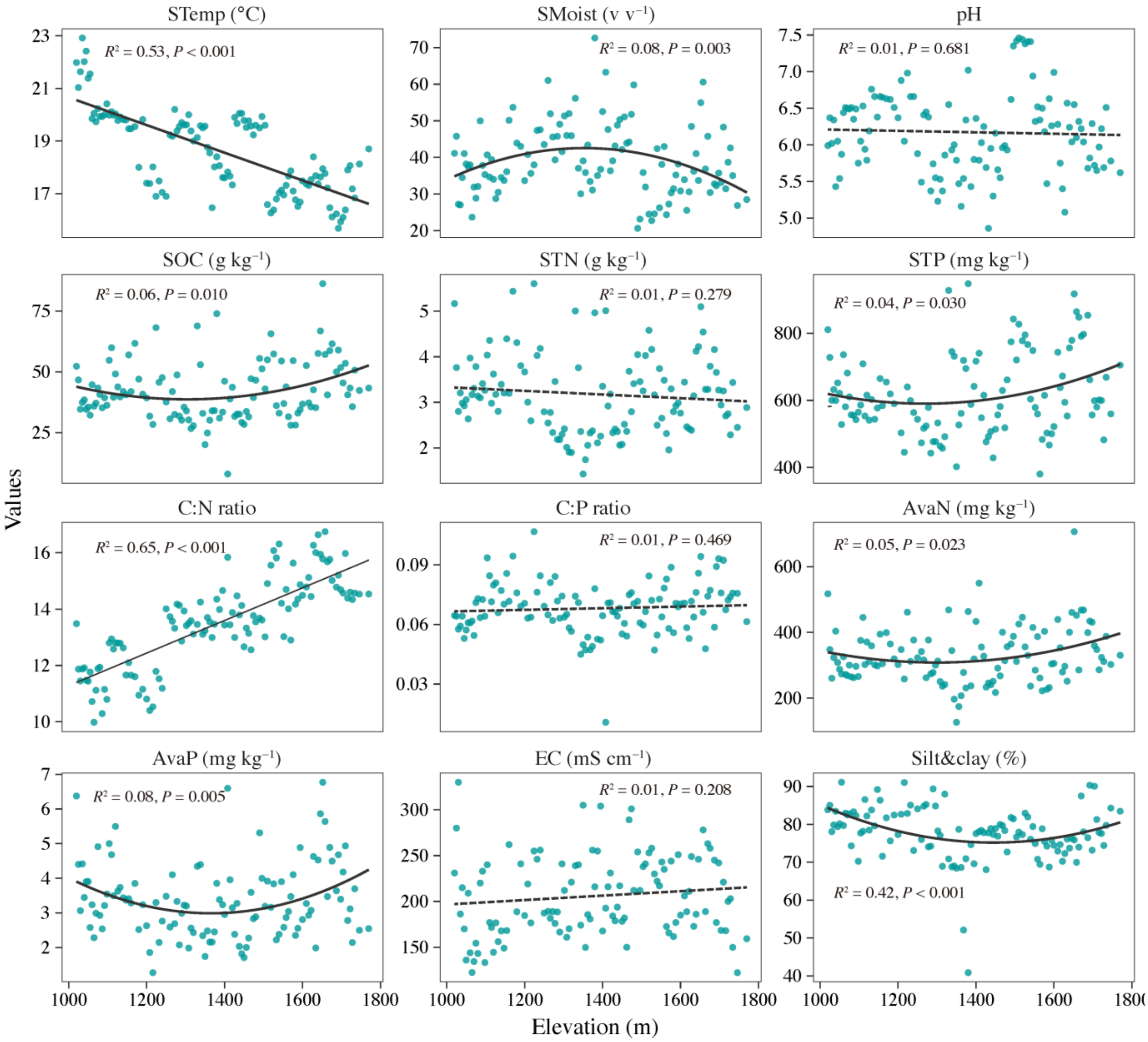

Figure S1. Variation of soil physicochemical factors along the altitudinal gradient. STemp, soil temperature; SMoist, soil moisture; SOC, soil organic carbon; STN, soil total nitrogen; STP, soil total phosphorus; C:N ratio, soil organic carbon to total nitrogen ratio; C:P ratio, soil organic carbon to total phosphorus; AvaN, soil available nitrogen; AvaP, soil available phosphorus; EC, soil electrical conductivity; Silt&clay, soil silt and clay. Solid and dashed lines denote significant and in-significant regressions.

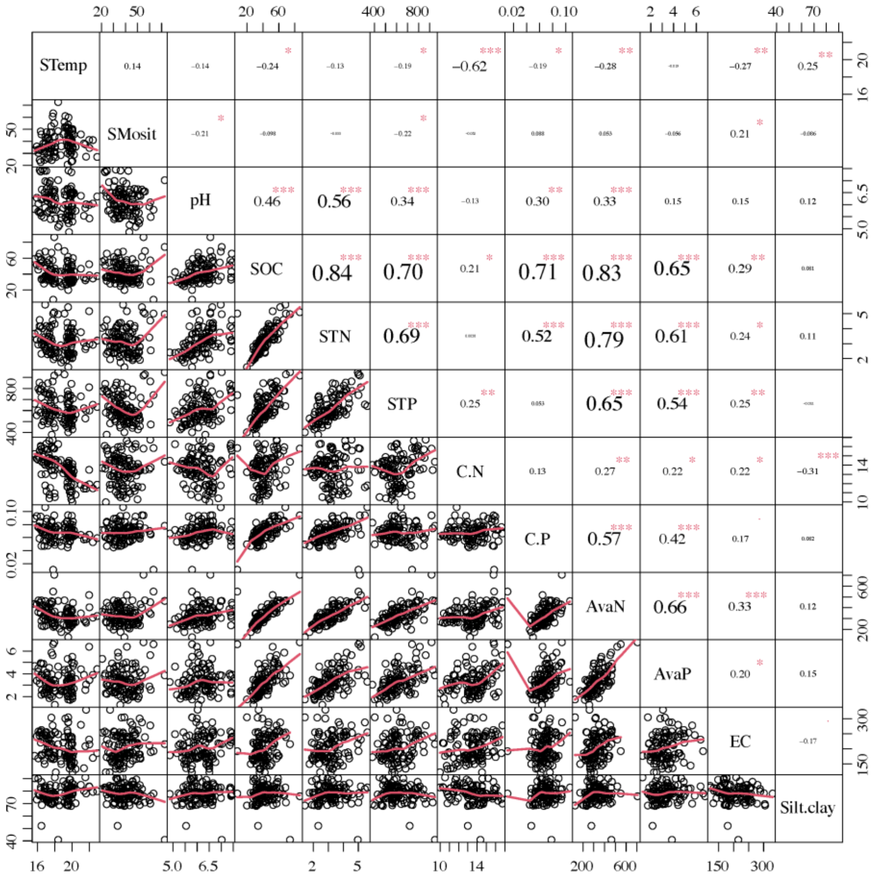

Figure S2. Spearman correlations between soil variables in the forest. **, significant at 0.01 level. STemp, soil temperature (℃); SMoist, soil moisture (v v^{-1}); SOC, soil organic carbon (g kg^{-1}); STN, soil total nitrogen (g kg^{-1}); STP, soil total phosphorus (mg kg^{-1}); C:N, ratio of soil organic carbon to total nitrogen; C:P, ratio of soil organic carbon to total phosphorus; AvaN, soil available nitrogen (mg kg^{-1}); AvaP, soil available phosphorus (mg kg^{-1}); EC, soil electrical conductivity (mS cm^{-1}); Silt&clay, soil silt and clay content (%).

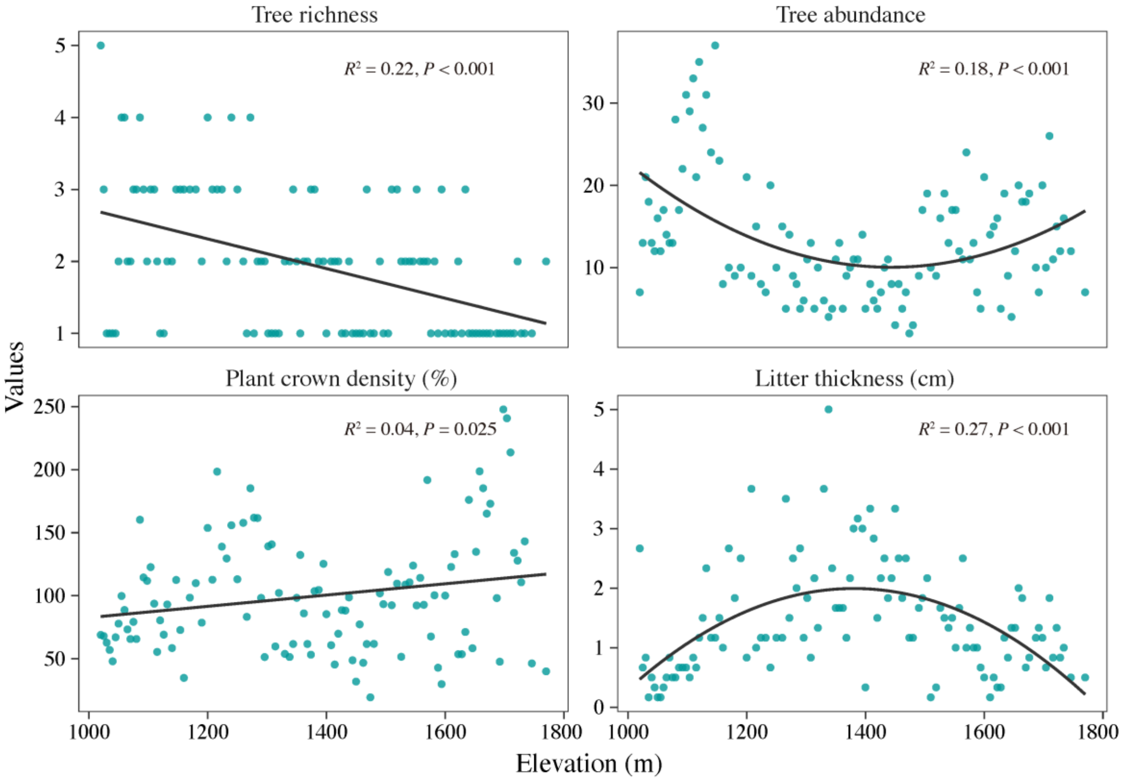

Figure S3. Variation of plant species richness, abundance, density, and litter thickness along the altitudinal gradient. Sodic lines denoted significant regressions.

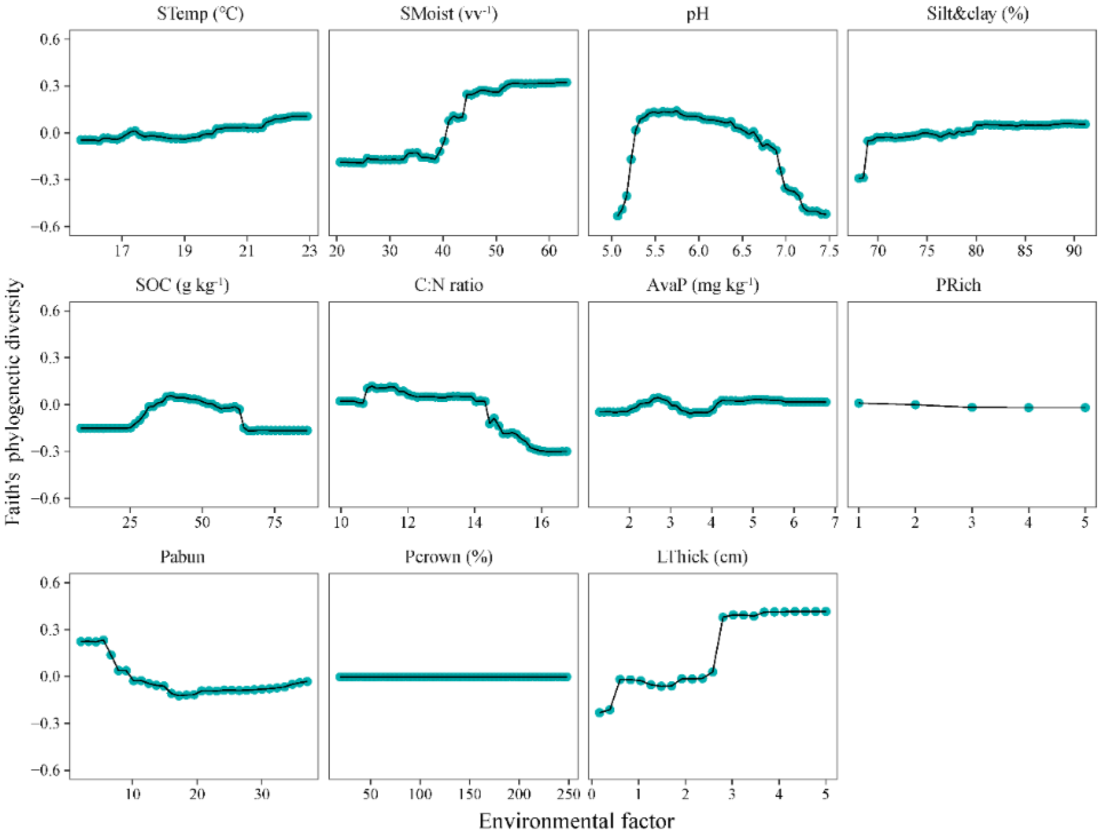

Figure S4. Partial dependence of plant, and soil properties on the Faith's phylogenetic diversity of soil bacteria using the Random forest modeling. STemp, soil temperature; SMoist, soil moisture; SOC, soil organic carbon; C:N ratio, soil organic carbon to total nitrogen ratio; AvaP, soil available phosphorus; PRich, species richness of plant; Pabun, abundance of plant; Pcrown, crown density of plant; LThick, litter thickness.

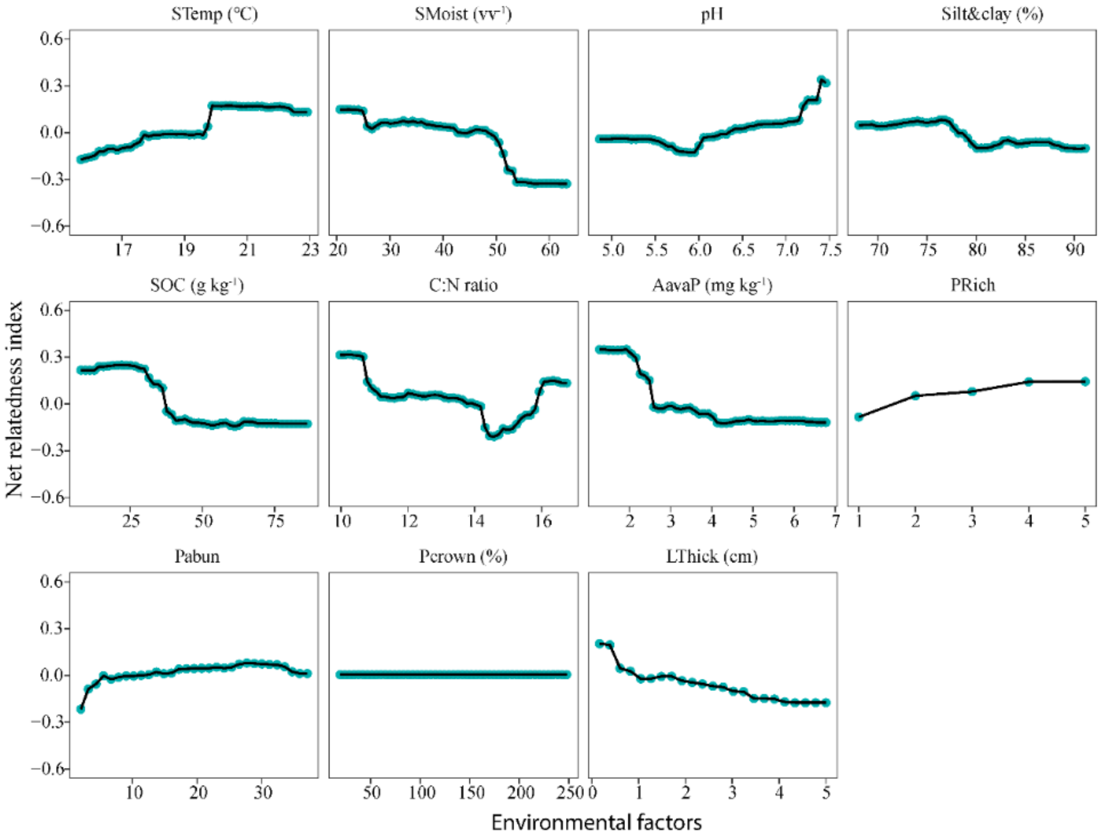

Figure S5. Partial dependence of plant, and soil properties on the net relatedness index of soil bacteria using the Random forest modeling. STemp, soil temperature; SMoist, soil moisture; SOC, soil organic carbon; C:N ratio, soil organic carbon to total nitrogen ratio; AvaP, soil available phosphorus; PRich, species richness of plant; Pabun, abundance of plant; Pcrown, crown density of plant; LThick, litter thickness.
